# Case Report: TNFα Antagonists Are an Effective Therapy in Cardiac Sarcoidosis

**DOI:** 10.3389/fcvm.2021.676407

**Published:** 2021-06-11

**Authors:** Julien Stievenart, Guillaume Le Guenno, Marc Ruivard, Virginie Rieu, Marc André, Vincent Grobost

**Affiliations:** ^1^Internal Medicine Department, Clermont-Ferrand University Hospital, Clermont-Ferrand, France; ^2^Université Clermont Auvergne, Clermont-Ferrand University Hospital, Inserm U1071, INRA USC2018, M2iSH, Clermont-Ferrand, France

**Keywords:** cardiac sarcoidosis, corticosteroids, immunosuppressive therapy, TNF antagonist, case series

## Abstract

**Introduction:** Cardiac sarcoidosis (CS) is a life-threatening disease in which clear recommendations are lacking. We report a case series of CS successfully treated by tumor necrosis factor (TNF)α antagonists.

**Methods:** We conducted a single-center retrospective study of our patients with CS treated by TNFα antagonists.

**Results:** Four cases (4/84, 4.7%) were found in our database. Mean age was 40 years (range 34–53 years), and all were Caucasian men. Mean follow-up was 54.75 months (range 25–115 months). All patients received corticosteroid therapy (CT) and immunosuppressive therapy (IT). TNFα antagonists (infliximab or adalimumab) were started after the first or second CS relapse under CT and IT. One patient experienced relapse under TNFα antagonists (isolated decreased left ventricular ejection) and responded to a shorter interval of TNFα antagonist infusion. CT was discontinued in three patients treated with TNFα antagonists without relapse or major cardiac events during follow-up. No serious adverse event occurred in our case series, possibly due to dose sparing and frequent arrest of CT.

**Conclusion:** TNFα antagonists were effective in refractory and/or relapsing CS treated by corticosteroids and/or immunosuppressive agents, without serious adverse events, and should be considered earlier in CS treatment scheme.

## Introduction

Sarcoidosis is a rare multisystemic granulomatous disease of unknown etiology, which most frequently involves the lungs, lymph nodes, skin, eyes, liver, and spleen (1). Cardiac sarcoidosis (CS) is a rare condition, with symptomatic cardiac features reported in 2.3–39% of patients with sarcoidosis (2, 3). Cardiac involvement in sarcoidosis ranges from 27 to 50% in morphological studies (4, 5). Although CS is rare, it can be a life-threatening condition, mainly with left ventricular (LV) systolic failure, ventricular arrhythmias, and atrioventricular conduction abnormalities, which can lead to disability or cardiac sudden death (6). Research, diagnosis, and management of CS have all seen great progress in the last few years (7, 8). Corticosteroid therapy (CT) remains the mainstay for CS, despite a lack of prospective controlled studies, and CT should be started early after CS diagnosis (9). Treatment is recommended based on clinician experience, expert opinion, and observational cohorts. To our knowledge, only two studies have investigated the impact of adjunctive immunosuppressive therapy (IT) on CS (10, 11).

In sarcoidosis, a key feature of granuloma is the interaction of ${\text{CD}}_{4}^{+}$ T cells with antigen-presenting cells to initiate and maintain the development of granuloma (1). ${\text{CD}}_{4}^{+}$ T cells differentiate into type 1 auxiliary T cells that secrete interleukin-2 and interferon-γ and increase production of TNFα, proinflammatory cytokines that amplify the cellular immune response (12). Therefore, TNFα appears to be an indicated therapy target. In severe or refractory disease, TNFα antagonists are effective in ocular (13), neurological (14), osseous (15), and pulmonary (16, 17), sarcoidosis. In CS, a few cases reports (18–22) and five cohort studies (23–27) have shown benefits of CT with or without IT in patients with severe and/or refractory cardiac involvement. Although there has been no randomized controlled study, some articles and expert opinions have suggested that TNFα antagonists for severe or refractory CS might be an option in case of CT or IT failure (28–30). We report our case series of CS treated by TNFα antagonists as adjunctive therapy.

## Methods

We conducted a single-center retrospective study of CS treated by TNFα antagonists using a systematic search of the Clermont-Ferrand CHRU CIM10 database in the Department of Internal Medicine, using code D868 “Sarcoidosis of other localizations and associated,” between January 2000 and January 2020. Inclusion criteria were CS diagnosis by endomyocardial biopsy positive for myocardial granuloma compatible with CS, or Heart Rhythm Society (HRS) criteria for probable CS (31), and use of TNFα antagonists in follow-up for CS. We collected data retrospectively concerning baseline demographic characteristics, previous diseases, sarcoidosis diagnosis, CS features, and follow-up. The critical endpoints were (1) clinical and/or imaging relapse defined as onset of a new CS manifestation or worsening of preexisting CS manifestation; (2) major adverse cardiovascular events [MACEs: defined as cardiac death, ventricular fibrillation, sustained ventricular tachycardia (sVT), and hospitalization for heart failure]; and (3) adverse medical or drug events. Treatment efficacy was defined by the absence of critical endpoint during follow-up with a CT dose of 5 mg or below. Treatment failure was defined by the presence of a critical endpoint during follow-up. Investigation of the data was approved by the local ethics committee *Comité de Protection des Personnes Sud-Est 6* (number 2020/CE 75). Patients gave oral consent for retrospective collection of their medical data.

## Results

**Figure 1:**
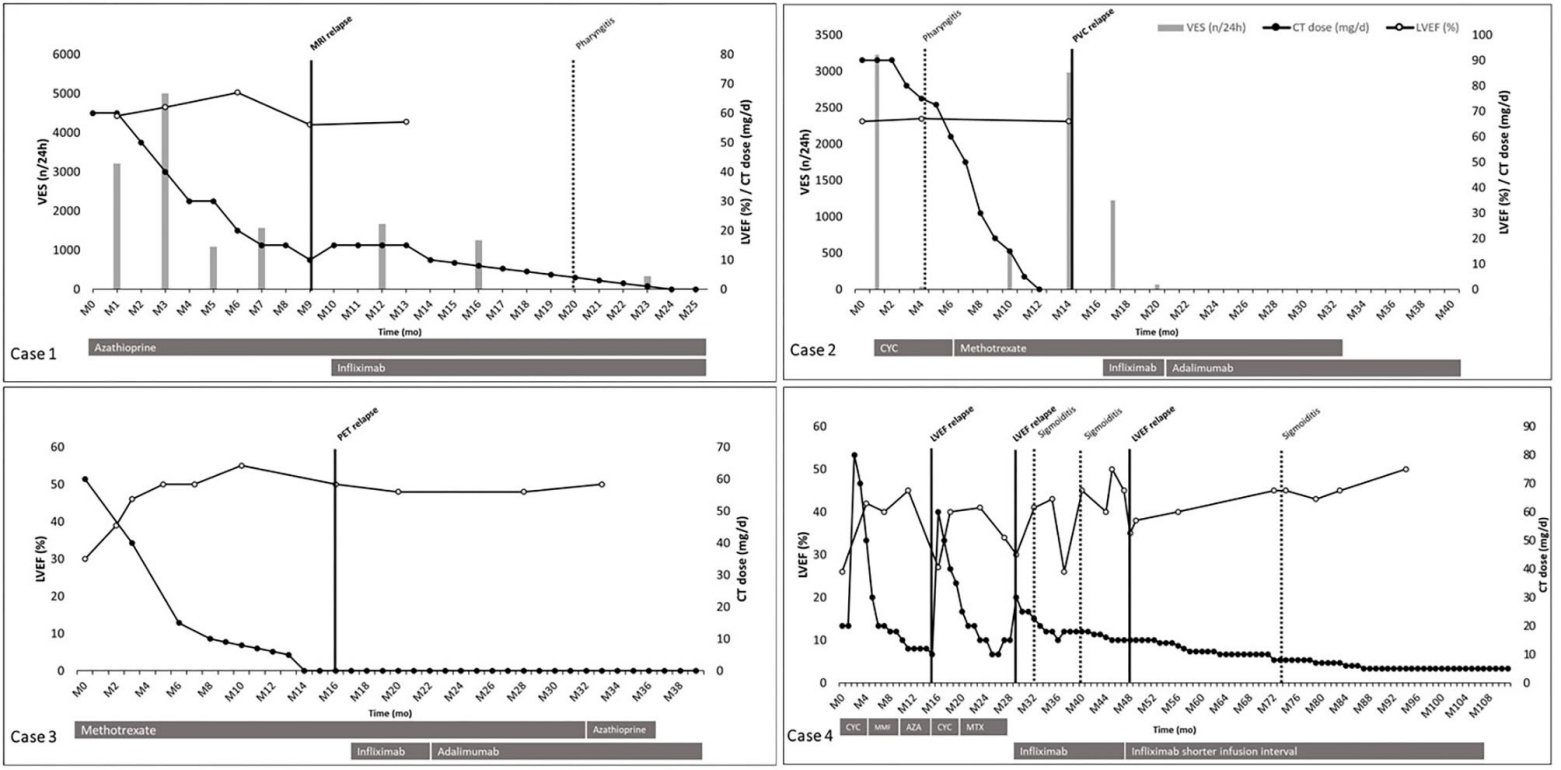
Patients' follow-up outcomes. Individual case presented from diagnosis (MO) to the end of follow-up for the study. Time in month on abscissa. Prednisone dose (mg/d), left ventricular ejection fraction (%), number of ventricular extrasystole (n/24 h) on Ordinate. Used therapies are represented in gray bars below each timeline. Black dots correspond to prednisone dose (mg/d). White dots correspond to left ventricular ejection fraction (%). Vertical gray bars correspond to number of ventricular extrasystole (n/24 h). Vertical black bars correspond to relapse. Vertical black dotted line corresponds to adverse events. Case 1: azathioprine was used 150 mg/d; infliximab was used 5 mg/kg at week 0 and 2, and every 4 weeks. Case 2: cyclophosphamide was used 1,000 mg/mo; methotrexate was used 15–20 mg/w; infliximab was used 5 mg/kg at week 0 and 2, and every 4 weeks; adalimumab was used 40 mg every 2 weeks, subcutaneously. Case 3: methotrexate was used 10–15 mg/w; azathioprine was used 100 mg/w; infliximab was used 5 mg/kg at week 0 and 2, and every 4 weeks; adalimumab was used 40 mg every 2 weeks, subcutaneously. Case 4: cyclophosphamide was used 1,000 mg/mo; mycophenolate mofetil was used 720 mg/d; azathioprine was used 150 mg/d; methotrexate was used 10–15 mg/w; infliximab was used 3 mg/kg at week 0 and 2, and every 8 weeks, then 5 mg/kg every 6 weeks. AZA, azathioprine; CI, corticosteroid therapy; CYC, cyclophosphamide; LVEF, left ventricular ejection fraction; MMF, mycophenolate mofetil; MRI, magnetic resonance imaging; MIX, methotrexate; PET, positron emission tomography; PVC, pre-mature ventricular contraction; VES, ventricular extrasystole.

We screened 84 patients and four met the inclusion criteria. Baseline characteristics are presented in Table 1 and follow-up outcomes in Figure 1. Mean age was 40 years (range 34–53 years), and all four patients were Caucasian men. Mean follow-up was 54.75 months (range 25–115 months). All four patients were treated with corticosteroids and immunosuppressive agents, including cyclophosphamide (CYC), azathioprine (AZA), methotrexate (MTX), and mycophenolate mofetil (MMF). Cardiac treatment was managed by the referent cardiologist. TNFα antagonists (infliximab and adalimumab) were given after the first or second CS relapse under CT and IT.

**Table 1:** Baseline characteristics of patients in our case series.

	**Case 1**	**Case 2**	**Case 3**	**Case 4**
**Age at sarcoidosis diagnosis, yr**	34	36	38	53
**Male**	+	+	+	+
**Caucasian**	+	+	+	+
**Habitus**				
Alcohol	0	0	0	0
Tobacco	+	+	+	+
**BMI (kg/m**^2^**)**	19.7	28.7	22.1	26.1
**Previous diseases**				
Cardiovascular disease	0	0	0	0
Diabetes mellitus	0	0	0	+
Hyperlipidaemia	0	0	0	0
**Organ involvement**				
Lungs	0	0	0	+
Lymph nodes	+	+	+	+
Skin	0	0	0	0
Ear, nose, throat	0	0	0	0
Eyes	0	+	0	0
Liver/spleen	+	+	0	0
Central nervous system	0	0	0	0
Peripheral nervous system	0	+	0	0
Kidney	0	0	0	0
**Pathological evidence of granuloma**	Salivary glands, lymph node	Lymph node	Lymph node	Lymph node
**Angiotensin-converting enzyme (UI/L**, ***N*** **<** **60)**	67	120	-	62
**Hypercalcaemia**	0	0	0	0
**Gammaglobulin level (g/L)**	12.5	16.3	9.8	12.1
**Time from diagnosis to CS diagnosis (months)**	2	1	0	6
**Initial evaluation**				
VES/24 h	3,213	3,227	0	0
sVT	0	0	0	0
nsVT	0	0	0	0
AV block	0	0	0	0
Bundle branch block	+	0	0	0
LVEF (%)	60	66	30	26
Wall motion abnormalities	0	0	+	+
NT-pro-BNP (ng/mL, *N* <150)	170	7	791	130
Troponin (pg/mL, *N* <0.015)	N	0.044	0.24	N
Cardiac PET	0	+	0	+
Cardiac LGE on MRI	+	0	+	0

*BMI, body mass index; LGE, Late gadolinium enhancement; LVEF, left ventricle ejection fraction; MRI, magnetic resonance imagery; N, Normal; nsVT, nonsustained ventricular tachycardia; PET, positron emission tomography; sVT, sustained ventricular tachycardia; VES, ventricular extrasystoles*.

All four patients experienced at least one relapse under IT alone (cases 2 and 3) or associated with CT (cases 1 and 4) before TNFα antagonists' initiation. In cases 2 and 3, relapses were a high number of premature ventricular contractions and a cardiac fluorodeoxyglucose positron emission tomography (FDG-PET) uptake, respectively. In each case, CT was tapered off within 12–14 months from initiation under MTX as immunosuppressive adjunctive therapy. Relapse occurred 2 months after CT discontinuation and led to TNFα antagonist initiation, without CT resumption. The chosen TNFα antagonist was infliximab at 5 mg/kg, at 0 and 2 weeks, and every 4 weeks. In each of those two cases, infliximab was changed for adalimumab 40 mg subcutaneously every 2 weeks, for the patient's personal convenience. These two patients did not show any evidence of relapse on a 19-month follow-up under adalimumab, without CT.

In cases 1 and 4, relapses were magnetic resonance imaging (MRI) late gadolinium enhancement with increasing angiotensin-converting enzyme and LVEF decreases, respectively. In case 1, MRI relapse occurred under prednisone at 10–15 mg/d and AZA, which led to infliximab initiation at 5 mg/kg, at 0 and 2 weeks, and every 4 weeks. CT was tapered to reach 5 mg within 9 months and was stopped within 14 months from infliximab initiation. In case 4, CS activity was defined by LVEF decrease. At diagnosis, CYC, mycophenolate mofetil and AZA permitted CT tapering to 10 mg/d of prednisone. First relapse/MACE (hospitalization for heart failure) was treated by increasing CT dose to 60 mg/d and new CYC pulses (1,000 mg/month for six pulses) relayed by MTX. Twelve months later, a second relapse/MACE occurred (hospitalization for heart failure) while the patient was under MTX and prednisone 15 mg/d. MTX was discontinued and infliximab was introduced, at 3 mg/kg, at 0 and 2 weeks, and every 8 weeks. Nineteen months later, under prednisone 15 mg/d and infliximab every 8 weeks, a third relapse/MACE (hospitalization for heart failure) occurred, leading to strengthening of the dose to 5 mg/kg and shortening of the infliximab infusion interval to 6 weeks. Thereafter, CT was slowly tapered down to reach 5 mg within 39 months after the third relapse, without any other relapse.

In those four patients (Table 2), a total of six relapses, considered as therapeutic failure, were recorded, in which three MACEs occurred in case 4. Five relapses occurred under standard IT associated or not with CT: three relapses under CT associated with IT, two relapses under IT alone. One relapse occurred under infliximab therapy in case 4 under infliximab 3 mg/kg every 8 weeks, successfully treated by increased dose of infliximab and shorter infusion interval. Adalimumab 40 mg every 2 weeks was used in two patients without any evidence of relapse or MACE.

**Table 2:** Relapses and MACEs in our patients.

**Events**	**Case 1**	**Case 2**	**Case 3**	**Case 4**	**Overall**
**Relapses**	1	1	1	3	6
IT					
CT associated	1	0	0	2	3 (50%)
IT alone	0	1	1	0	2 (33%)
TNFα antagonists	0	0	0	1	1 (16%)
**MACEs**	0	0	0	3	3
IT					
CT associated	0	0	0	2	2 (66%)
IT alone	0	0	0	0	0
TNFα antagonists	0	0	0	1	1 (33%)
**Overall events**	1	1	1	3	6

*CT, corticosteroid therapy; IT, immunosuppressive therapy; MACEs, major adverse cardiovascular events; TNFα, tumor necrosis factor alpha. In case 4, MACEs were also considered as relapses*.

Standard IT, with or without CT associated, failed five times whereas TNFα antagonists failed once. TNFα antagonists demonstrated efficacy defined by the absence of critical endpoint during follow-up with a CT dose of 5 mg or below in all cases, with a follow-up under TNFα antagonists of 16–80 months. In case 4, TNFα antagonists failed to demonstrate efficacy with one relapse under prednisone 15 mg/d and infliximab 3 mg/kg every 8 weeks, but succeeded to demonstrate efficacy at 5 mg/kg every 6 weeks.

Five infectious adverse events were retrieved in cases 1, 2, and 4, consisting in a pharyngitis under prednisone 3 mg/d, azathioprine 150 mg/d, and infliximab 300 mg every 4 weeks in case 1; a pharyngitis under prednisone 75 mg/d, CYC 1,000 mg/month in case 2; and three sigmoiditis treated by oral antibiotics under prednisone 22.5–8 mg/d and infliximab in case 4. No drug-related adverse event or serious adverse event was recorded.

## Discussion

This case series included all patients treated with the TNFα antagonist (infliximab or adalimumab) for CS in our center. TNFα antagonists show an effectiveness in all patients as rescue treatment in CS relapsing under IT and/or CT, in long and close follow-up without serious infectious or drug-related adverse events. As reported by Ballul et al. (10), our patients were Caucasian men who relapsed even with IT, although they suggested that IT associated with CT may reduce the relapse rate. Only one patient experienced relapse under TNFα antagonists, but without other IT, and responded to a stronger dose and shorter TNFα antagonist infusion interval. CT was discontinued in three patients treated with TNFα antagonists without relapse or MACEs during long-term follow-up and only one patient had a long-term low-dose prednisone of 5 mg/d. Adalimumab and infliximab were the chosen TNFα antagonists in our patients, because of their effectiveness in extra-cardiac sarcoidosis (32) and because of the convenience of subcutaneous administration of adalimumab and reassuring data on switching infliximab to adalimumab (33). In our patients, adalimumab did not show any evidence of inferiority compared to infliximab since we did not record any relapse or adverse event under this therapy. In two patients, CT was discontinued before TNFα antagonists' initiation, and relapses were treated with infliximab only, without resumption of CT.

Standard therapy including IT and/or CT failed in all our cases, whereas one patient relapsed under infliximab but succeeded with a stronger dose and shorter infusion interval. In this case, infliximab dose was lower (3 mg/kg) and interval infusion was greater (8 weeks) than the other cases, due to referral physician decision and lack of standard protocol in CS. Moreover, this patient received infliximab 28 months after CS diagnosis with already two relapses. This remark raises the question of early “optimal” treatment in CS in order to avoid myocardial scar formation after each relapse, which are probably not responsive to intensive treatment. In three out of four cases, IT was used in combination with TNFα antagonists, as corticosteroid-sparing agents and as demonstrated by Ballul et al. (10) as CS specific treatment. This attitude should be highlighted because of the effectiveness of IT in CS, especially in relapsing or in CT-dependent patients. Although all our cases failed under IT alone or associated with CT, IT utility, when used aside from TNFα antagonists, remain unclear in CS, and further studies might be interesting.

No serious infectious or drug related adverse event occurred in our case series, possibly due to dose sparing and frequent stopping of CT. In fact, 3 out of 4 cases discontinued CT 11–23 months after CS diagnosis and last patient had a 49 months follow-up under the threshold of prednisone 10 mg.

Until 2019, there were no randomized trials on TNFα antagonists in CS, and only a few cases reports or series had shown the potential benefit of TNFα antagonists (17–22). The ATTACH (Anti-TNF Therapy Against Congestive Heart Failure) trial (34) found worsening of heart failure in patients treated by high-dose infliximab for congestive heart failure with other causes than sarcoidosis limiting its use in cardiac inflammatory conditions such as CS. However, Drent et al. (29) have suggested using TNFα antagonists, especially infliximab, if there is no response to conventional treatment, in the presence of active CS and if CS is identified as the only cause of heart failure. Adler et al. conducted a systematic review of efficacy and safety of TNFα antagonists in sarcoidosis (32). They showed similar rates of adverse events (88.2 vs. 91%) and severe adverse events (18.5 vs. 14.8%) in five randomized control trials comparing TNFα antagonists with placebo. Rates of malignancy and death were comparable between groups (1.1 vs. 0.8% for malignancies and 1.1 vs. 1.6% for deaths, TNFα compared to placebo).

In September 2019, Rosenthal et al. (25) recommended adalimumab as second-line therapy after high-dose prednisolone (>30 mg/day for 4–8 weeks) and MTX (20 mg/week) if patients experienced clinical relapse, did not achieve FDG-PET remission, or experienced adverse effects of previous immunosuppression. This retrospective, single center study included 29 patients with CS according to the 2017 criteria of the Japanese Cardiology Society. The study found that discontinuation of immunosuppression was significantly associated with FDG-PET SUV increase and VT recurrence, and steroid-sparing immunosuppressive agents such as MTX or adalimumab were effective in suppressing inflammation in CS. In November 2019, Harper et al. (24) reported 36 patients treated by infliximab for CS refractory to conventional treatment. Twenty-four (66%) patients were categorized as “responders” after infliximab initiation in at least one of the three outcomes categories (steroid-sparing dose, LVEF, and dysrhythmia). Similar to our case series concerning sparing the dose of CT, Baker et al. (23) reported 20 patients with CS treated by TNFα antagonists. Seventeen patients had complete resolution of disease imaging activity within 12 months, and LVEF did not change (44 vs. 47%). TNFα antagonists permitted a decrease in mean dose of CT (23–4 mg/day) at 6 months and complete discontinuation of CT after a mean of 9 months of treatment with TNFα antagonists. In 2020, Gilotra et al. (26) reported 38 patients with CS treated with TNFα antagonists (infliximab or adalimumab), with a significant decrease in CT dose within 6 or 12 months of treatment, and CT discontinuation in 10 out of 38 patients. The increase in LVEF after treatment was not significant (45 before vs. 47% after treatment), and four (11%) patients suffered from infection as an adverse effect of IT. Finally, in 2021, Bakker et al. (27) reported 22 patients with CS treated by infliximab, with a mean follow-up of 18.9 months. Eighteen patients (82%) were classified as “responders,” with a significant decrease in myocardial SUVmax on FDG-PET and a significant increase in LVEF after TNFα antagonist initiation.

Comparing our case series to these recent data (Table 3), we found similar indications of TNFα antagonists use in CS suffering patients, particularly in patients with relapse occurring under IT. All studies described were retrospective. Mean follow-up in our series was higher than in the other series. Our study endpoints were relapses and MACEs as defined in our method whereas the other studies chose specific parameters such as prednisone dose, LVEF, myocardial FDG-PET uptake and dysrhythmias. CT and IT use at TNFα antagonists' initiation were similar throughout studies, as were CT mean doses and tapering schemes after TNFα antagonist initiation. Most used IT was methotrexate. Most used TNFα antagonists were infliximab at 5 mg/kg at week 0 and 2, and every 4 weeks and subcutaneous adalimumab 40 mg every 2 weeks. In two studies (24, 35), 66% (24/36) and 82% (18/22) TNFα antagonist's treated patients were classified as “responders.” Imaging endpoints were chosen in three studies (23, 25, 27) with good outcomes on disease activity on myocardial FDG-PET uptake. Data on CT discontinuation after TNFα antagonists' initiation were scarce, but studies found significant decrease in CT dose in the follow-up under TNFα antagonists (24, 26). Data on risk of worsening heart failure under TNFα antagonists were reassuring in two studies, with a global LVEF stability before and after treatment initiation (26) and significant LVEF increase from 45 to 55% (27). Nonetheless, a specific attention toward worsening heart failure under TNFα antagonists should be mentioned, as four patients experienced worsening in LVEF in Gilotra et al. cohort (26), especially in patients with severely impaired pre-treatment LVEF (20–35%). A few major drugs associated adverse events were recorded, such as sepsis, *Cryptococcus* infection, aseptic meningitis, *C. difficile* diarrhea, hepatitis, and allergic reaction during infusion. In overall cohort studies, 23 adverse events in 139 TNFα antagonists treated patients were retrieved. All these data taken together emphasize TNFα antagonists' efficacy and safety but highlight the lack of homogeneity in study design and prevent us from drawing any clear recommendations.

**Table 3:** Outcomes in recent studies upon TNFα antagonists' efficiency in CS.

**Endpoints**	**Baker et al. (23)**	**Harper et al. (24)**	**Rosenthal et al. (25)**	**Gilotra et al. (26)**	**Bakker et al. (27)**	**Our series**
TNFα antagonist treated of patients (*n*)	20	36	19	38	22	4
Mean follow up (mo)	Data not available	<12	49.2	15.9	18.9	54.7
Evaluation criteria	Prednisone dose, LVEF	LVEF, dysrhythmias, prednisone dose	FDG-PET uptake	Clinical composite criteria^†^, FDG-PET uptake	FDG-PET uptake, device interrogation, TTE, biomarker, treatment dose	Relapse, MACEs
CT and IT used at TNFα antagonist introduction, number of treated patients (*n*)	MTX, *n* = 20 Prednisone and MTX, *n* = 13	Prednisone 10–30 mg/d, *n* = 35 MTX, *n* = 25 LEF, *n* = 9 AZA, *n* = 1 HCQ, *n* = 2	Data not available	Prednisone alone, *n* = 2 Prednisone and MMF (mean dose 2 g/d), *n* = 16 Prednisone and MTX (mean dose 16 mg/w), *n* = 11 Prednisone and AZA (mean dose 171 mg/d), *n* = 8	Prednisone, *n* = 12 MTX, *n* = 10 AZA, *n* = 6 MMF, *n* = 3 HCQ, *n* = 1	Prednisone 0–20 mg/d (*n* = 2) and AZA 100–150 mg/d (*n* = 2)or MTX 10–20 mg/w (*n* = 2)
Mean time from CS diagnosis to TNFα antagonist introduction (mo)	16	Data not available	Data not available	16.1	22.8	17.25 (9–28)
TNFα antagonist treatment scheme, number of treated patients (*n*)	IFX (data not available), *n* = 10 ADA (data not available), *n* = 10 GOL (data not available), *n* = 1	IFX 5–10 mg/kg every 4–8 weeks, *n* = 36	ADA 40 mg every 2 weeks *n* = 19	IFX 3–10 mg/kg (interval not shown), *n* = 30 ADA 40 mg every 2 weeks, *n* = 8	IFX 5 mg/kg week 0 and 2, and every 4 weeks	IFX 3–5 mg/kg every 4–8 weeks *n*=4 ADA 40 mg every 2 weeks *n* = 2
Prednisone tapering scheme after TNFα antagonist initiation	Mean of 23 mg tapered to mean of 4 mg over 6 months	Mean of 20 mg/d tapered to mean of 5 mg/d over 12 months	Data not available	21.7 ± 17.5 mg/d tapered to 7.3 ± 7.3 mg/d over 12 months	Data not available	0–30 mg/d tapered to 0–5mg/d Discontinuation before (*n* = 2) Discontinuation 13 months after (*n* = 1)
CT discontinuation after TNFα antagonist initiation (*n*, %)	Data not available	Data not available	Data not available	10 (26%)	Data not available	*n* = 1 (25%), 13 months after
Adverse events under TNFα antagonist (*n*)	0	6	1	8	5	3
Mean follow-up under TNFα antagonists (mo)	12	<12	Data not available	<12	18.9	33.8 (16–80)
Relapse (*n*)	Data not available^*^	Data not available^*^	Data not available^*^	Data not available^*^	Data not available^*^	6
MACE (*n*)	Data not available^*^	Data not available^*^	Data not available^*^	Data not available^*^	Data not available^*^	3
Relapse under TNF (*n*, %)	Data not available^*^	8 (22%)	Data not available^*^	Data not available^*^	2 (9%)	1 (25%)
MACE under TNF (*n*, %)	Data not available^*^	Data not available^*^	Data not available^*^	Data not available^*^	Data not available^*^	1 (25%)

† *Incidence of ventricular arrhythmias, worsening heart failure, heart transplantation, left ventricular assist device implantation and death from any cause*.

* *Relapse and MACEs were not defined in each study's methodology. ADA, adalimumab; AZA, azathioprine; CT, corticosteroid therapy; GOL, golimumab; HCQ, hydroxychloroquine; IFX, infliximab; LVEF, left ventricular ejection fraction; MACE, major adverse cardiac event; MMF, mycophenolate mofetil; MTX, methotrexate; TNFα, tumor necrosis factor alpha; TTE, transthoracic echocardiography*.

In 2021, Kouranos et al. published a state-of-the-art review on CS (35), emphasizing early use of CT in CS but without established scheme. Also, initial dosage remain unclear. Use of IT such as methotrexate is mentioned as of interest in avoiding steroid related adverse events and dysrhythmias such as ventricular extra systole and non-sustained ventricular tachycardia. Nonetheless, duration and dose remain unknown in this specific condition. Unfortunately, TNFα antagonists were not mentioned as possible part of CS treatment, including as rescue treatment in severe of refractory CS to conventional treatment.

TNFα antagonists are effective in CS cases that failed or relapsed under conventional IT, including CT or non-biological IT, and represent a good steroid-sparing therapy with an overall good tolerance profile in our case study and in the literature data.

Our case study had some limitations. Only four patients received TNFα antagonists for CS at our institution, which limited interpretation of the usefulness of this medication. Moreover, evolution of each patient could be attributed to CS natural course, due to the lack of a control group. The retrospective nature of the study meant that our patients had different treatment regimens that were adapted by referral physicians to the specific condition of each patient. The initial starting dose of CT was 1 mg/kg/day in each case, which was higher than some proposed regimens in the literature but without long-term maintenance dose of CT because of the use of IT and TNFα antagonists. In retrospective studies, CT schemes varied a lot as shown in Sadek et al. systematic review on CT in CS (9), probably due to physicians' decision toward each patient conditions and retrospective design of most of the studies.

In order to clarify TNFα antagonists' position in the treatment scheme of CS, further studies are needed, with control groups, standardized treatment schemes on CT and IT use, and strong clinical endpoints such as relapse or MACEs. However, in the absence of such studies, we recommend TNFα antagonists use early in severe or relapsing CS, because of the life-threatening issues in this specific condition, and to avoid long and/or high CT cumulative exposure in frequently young people.

## Conclusion

We reported a case series of four patients successfully treated by TNFα antagonists for relapsing CS under CT and IT, with good long-term outcomes regarding relapse rate, steroid-sparing dose and adverse drug events. Treatment regimens and standardized approaches are lacking in CS, showing that a tailored approach is needed for each patient to achieve remission, but with the possibility of TNFα antagonists use in case of severe or relapsing CS under IT and/or CT.

## Data Availability Statement

The original contributions presented in the study are included in the article/supplementary material, further inquiries can be directed to the corresponding author.

## Ethics Statement

Investigation of the data was approved by the local ethics committee Comité de Protection des Personnes Sud-Est 6 (number 2020/CE 75). The patients/participants provided their oral and written informed consent to participate in this study, and oral consent to publication of their anonymous data.

## Author Contributions

All authors listed have made a substantial, direct and intellectual contribution to the work, and approved it for publication.

## Conflict of Interest

The authors declare that the research was conducted in the absence of any commercial or financial relationships that could be construed as a potential conflict of interest.

## Abbreviations

AZA: Azathioprine
CS: Cardiac sarcoidosis
CT: Corticosteroid therapy
CYC: Cyclophosphamide
FDG: Fluorodeoxyglucose
HRS: Heart Rhythm Society
IT: Immunosuppressive therapy
LVEF: Left ventricular ejection fraction
MACEs: Major adverse cardiovascular events
MMF: Mycophenolate mofetil
MRI: Magnetic resonance imaging
MTX: Methotrexate
PET: Positron emission tomography
VT: Ventricular tachycardia
sVT: sustained ventricular tachycardia
TNFα: Tumor necrosis factor α.
